# Adverse childhood experiences and binge-eating disorder in early adolescents

**DOI:** 10.1186/s40337-022-00682-y

**Published:** 2022-11-16

**Authors:** Jonathan Chu, Julia H. Raney, Kyle T. Ganson, Kelsey Wu, Ananya Rupanagunta, Alexander Testa, Dylan B. Jackson, Stuart B. Murray, Jason M. Nagata

**Affiliations:** 1 Department of Pediatrics, University of California, 550 16th Street, 4th Floor, Box 0110, San Francisco, CA 94158 USA; 2 Factor-Inwentash Faculty of Social Work, University of Toronto, 246 Bloor Street W, Toronto, ON M5S 1V4 Canada; 3 Department of Management, Policy and Community Health, University of Texas Health Science Center at Houston, 7000 Fannin St, Houston, TX 77030 USA; 4 Department of Population, Family, and Reproductive Health, Johns Hopkins Bloomberg School of Public Health, Johns Hopkins University, 615 N Wolfe St, Baltimore, MD 21205 USA; 5 Department of Psychiatry and Behavioral Sciences, University of Southern California, 2250 Alcazar Street, Suite 2200, Los Angeles, CA 90033 USA

**Keywords:** Adolescent health, Adverse childhood experiences (ACEs), Binge-eating disorder

## Abstract

**Background:**

Adverse childhood experiences (ACEs) are common and linked to negative health outcomes. Previous studies have found associations between ACEs and binge-eating disorder (BED), though they have mainly focused on adults and use cross-sectional data. The objective of this study was to examine the associations between ACEs and BED in a large, national cohort of 9–14-year-old early adolescents in the US.

**Methods:**

We analyzed prospective cohort data from the Adolescent Brain Cognitive Development (ABCD) Study (N = 10,145, 2016–2020). Logistic regression analyses were used to determine the associations between self-reported ACEs and BED based on the Kiddie Schedule for Affective Disorders and Schizophrenia at two-year follow-up, adjusting for sex, race/ethnicity, baseline household income, parental education, site, and baseline binge-eating disorder.

**Results:**

In the sample, (49% female, 46% racial/ethnic minority), 82.8% of adolescents reported at least one ACE and 1.2% had a diagnosis of BED at two-year follow-up. The mean number of ACEs was higher in those with a diagnosis of BED compared to those without (2.6 ± 0.14 vs 1.7 ± 0.02). The association between number of ACEs and BED in general had a dose–response relationship. One ACE (adjusted odds ratio [aOR] 3.48, 95% confidence interval [CI] 1.11–10.89), two ACEs (aOR 3.88, 95% CI 1.28–11.74), and three or more ACEs (aOR 8.94, 95% CI 3.01–26.54) were all associated with higher odds of BED at two-year follow-up. When stratified by types of ACEs, history of household mental illness (aOR 2.18, 95% 1.31–3.63), household violence (aOR 2.43, 95% CI 1.42–4.15), and criminal household member (aOR 2.14, 95% CI 1.23–3.73) were most associated with BED at two-year follow-up.

**Conclusions:**

Children and adolescents who have experienced ACEs, particularly household challenges, have higher odds of developing BED. Clinicians may consider screening for ACEs and providing trauma-focused care when evaluating patients for BED.

**Supplementary Information:**

The online version contains supplementary material available at 10.1186/s40337-022-00682-y.

## Plain English summary

Adverse childhood experiences (ACEs) are common among adolescents and a significant public health concern given their associations with various health outcomes later in life. The current study examined the associations between ACEs and binge-eating disorder (BED) among early adolescents using a large, national sample in the United States. Participants with three or more ACEs had nearly nine times higher odds of developing BED at two-year follow-up. Of the different types of ACEs, household mental illness, household violence, and having a criminal household member were most strongly associated with BED. Screening for ACEs when evaluating adolescents for BED should be considered, and clinicians should provide trauma-informed care for adolescents with BED.

**Supplementary Information:**

The online version contains supplementary material available at 10.1186/s40337-022-00682-y.

## Background

Adverse childhood experiences (ACEs), defined as potentially traumatic abuse, neglect, and household challenges during childhood and adolescence, can have detrimental effects on health and wellbeing [1]. The prevalence of ACEs is high, with the Centers for Disease Control and Prevention (CDC) reporting nearly 61% of adults having experienced at least one type of ACE before age 18, and one in six adults having experienced four or more types of ACEs [2, 3]. Accumulating ACEs has been particularly linked to deleterious medical, mental health, and social outcomes [4, 5]. Given the high prevalence and ongoing public health burden of ACEs, additional studies and interventions are necessary to both elucidate and reduce their impact, as proposed by the American Academy of Pediatrics [6, 7].

A growing body of research has described relationships between ACEs and eating disorders (EDs). EDs, which include but are not limited to diagnoses such as anorexia nervosa, bulimia nervosa, and binge-eating disorder (BED), are complex psychiatric conditions associated with significant distress, medical comorbidities, and high mortality rates [8–10]. Previous studies have demonstrated associations between childhood trauma and EDs and disordered eating behaviors in adulthood [11–15]. For example, a national survey of adult men and women found higher rates of post-traumatic stress disorder (PTSD), sub-threshold PTSD, and exposure to any type of trauma among those with EDs, particularly BED [15]. In one cross-sectional, nationally representative sample of U.S. young adults (mean age 22 years), those with multi-type childhood maltreatment, reported a two-fold increase in the odds of binge-eating symptoms [13]. Another cross-sectional study using a clinical sample of adult patients receiving eating disorder treatment reported higher levels of ACEs among all patients with eating disorders, and those with BED had even higher levels of ACEs compared to those with anorexia nervosa, restricting subtype [16].

Despite bourgeoning research describing the relationship between ACEs and EDs, several important gaps remain. Because EDs typically develop between late adolescence and young adulthood, prior research has mainly focused adults and young adults [17, 18]. Recent studies have demonstrated BED, the most common eating disorder characterized by frequent episodes of food overconsumption, loss of control feelings of shame, and marked distress with episodes [19], often first presents in middle childhood and early adolescence [20]. Middle childhood and early adolescence are critical periods of psychosocial development vulnerable to the establish of health-related behaviors [21, 22]. Thus, early prevention and detection of BED in the early adolescent time period is crucial. Meta-analyses have found an overall estimated prevalence of 1.3% BED and 3.0% subclinical BED in children and adolescents [23]. Given links between BED and health outcomes such as obesity and depression [24, 25], both prevalent and high incidence conditions during adolescence [26, 27], further investigation is critical to understanding how binge-eating disorder develops in children.

The objective of this study was to determine the associations between number of ACEs and BED in a population-based, demographically diverse cohort of 9–14 year-old early adolescents and to determine which types of ACEs (i.e., abuse, neglect, household challenges) were most strongly associated with BED given the little research on individual ACEs and BED. We hypothesized that a greater number of ACEs would be associated with greater odds of BED in early adolescence.

## Methods

### Study population

We analyzed prospective data from the Adolescent Brain Cognitive Development (ABCD) Study, a longitudinal study of brain development and health across adolescence in 11,875 children recruited from 21 sites around the U.S. To recruit a sample representative of U.S. diversity, the ABCD study implemented epidemiologically-informed strategies largely through school systems and considering sociodemographic factors. Additional details are described elsewhere [28]. Data analyzed are from the ABCD 4.0 release for the baseline (2016–2018, 9–10-years-old) and two-year-follow-up (2018–2020, 10–14 years-old) assessments. Participants with missing data for ACEs during the study or BED at baseline and two-year follow-up were excluded, yielding the total sample of 10,145. We used Gaussian normal regression imputation to impute missing data for participants with missing confounder data. Centralized institutional review board (IRB) approval was obtained from the University of California, San Diego. Study sites obtained approval from their respective IRBs, caregivers provided written informed consent, and each child provided written assent. Data used in this study were obtained from the ABCD Study (https://abcdstudy.org), held in the NIMH Data Archive (NDA).

### Exposure: ACE score

ACE score was determined through parent and adolescent responses in the baseline (2016–2018), one-year follow-up (2017–2019), and the first half of two-year follow-up (2018–2019) surveys. The ABCD study assesses nine out of ten ACEs reflecting the items in the original CDC-Kaiser ACE study [4]; emotional abuse was not included as it was not evaluated in the ABCD study. A “yes” response to any of the following nine ACEs at any time point was counted as one point: physical abuse, sexual abuse, household violence, household mental illness, substance abuse in the household, divorce/separation, criminal household member, emotional neglect, and physical neglect. These points were totaled to yield an ACE score. Further details regarding the coding of ACE variables are presented in Additional file 1: Table 1. Previous literature has demonstrated increased deleterious outcomes with accumulated ACEs [4, 5].

### Outcome: binge-eating disorder

BED was assessed at the two-year follow-up through the Kiddie Schedule for Affective Disorders and Schizophrenia (KSADS-5), a computerized tool for categorizing child and adolescent mental health concerns based on the DSM-5 [19, 29]. Parents/caregivers completed all modules of the KSADS-5 to frequency, duration, characteristics of their child’s binge eating as well as associated distress. Using the KSADS-5 computerized scoring system, responses to the interview questions were extrapolated into their respective diagnosis from reported symptoms corresponding to the DSM-5 [19].

## Confounders

We selected potential sociodemographic confounders for the association between screen time and behavioral disorders based on previous literature and theory [30]. Age (years), sex (female, male), race/ethnicity (White, Latino/Hispanic, Black, Asian, Native American, other), household income (U.S. dollars, six categories: Less than $25,000, $25,000 through $49,999, $50,000 through $74,999, $75,000 through $99,999, $100,000 through $199,999, $200,000 and greater), and highest parent education (high school or less vs. college or more) were based on baseline parents’ self-report. ABCD Study site was included as a confounder to adjust for potential regional variation. Diagnosis of binge-eating disorder at baseline was further included as a confounder.

### Statistical analysis

Multiple logistic regression analyses were conducted in 2022 using Stata 15.1 (StataCorp, College Station, TX) to estimate associations between ACEs (exposure variable) and BED at two-year-follow-up (outcome variable), adjusting for confounders listed above. ACEs were analyzed as a cumulative score and individually in separate models. Propensity weights were applied to yield representative estimates based on the American Community Survey from the US Census [31]. Given previously studied relationships between trauma, BED, and other psychiatric disorders [15, 32, 33], additional sensitivity analyses were performed adjusting for baseline major depressive disorder, generalized anxiety disorder, and PTSD, with all findings unchanged.

## Results

The descriptive characteristics of the 10,145 participants are shown in Table 1. The analytic sample was approximately matched by sex (49% female) and was racially and ethnically diverse (46% non-White). Four out of five participants reported a history of at least one ACE, and 9.71% reported four or more ACEs. The prevalence of BED at two-year follow-up was 1.16%.

**Table 1:** Sociodemographic, adverse childhood experiences, and binge-eating disorder of 10,145 adolescent brain cognitive development (ABCD) study participants

Sociodemographic characteristics (baseline)	Mean (SD)/%
Age (years)	9.9 (0.6)
Sex, n (%)	
Female	48.90
Male	51.10
Race/ethnicity (%)	
White	54.00
Latino/Hispanic	19.80
Black	16.10
Asian	5.40
Native American	3.20
Other	1.50
Household income (%)	
Less than $25,000	16.20
$25,000 through $49,999	20.50
$50,000 through $74,999	18.30
$75,000 through $99,999	16.50
$100,000 through $199,999	21.80
$200,000 and greater	6.80
Parent with college education or more (%)	81.60
Number of ACEs*	
0	17.20
1	31.20
2	26.40
3 +	25.21
ACE subtype present	
Physical abuse	1.07
Sexual abuse	1.22
Emotional neglect	1.57
Physical neglect	7.19
Household substance use	40.77
Household divorce or separation	17.24
Household mental illness	31.85
Household violence	55.59
Criminal household member	15.32
Binge-eating disorder (baseline)	0.57
Binge-eating disorder (two-year follow-up)	1.16

Propensity weights were applied to yield nationally representative estimates based on the American Community Survey from the US Census. SD = standard deviation

ACEs = Adverse Childhood Experiences

**Fig. 1:**
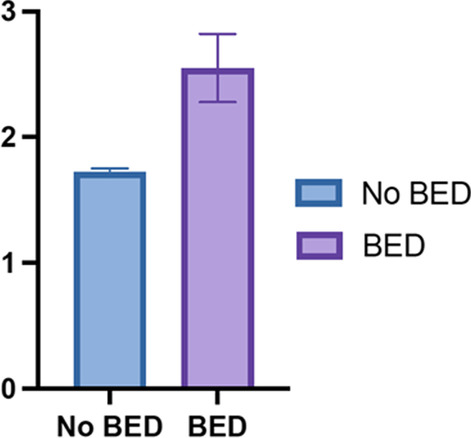
Mean number of ACEs among participants with and without BED

Figure 1 shows the mean number of ACEs in participants with and without a diagnosis of BED (2.6 ± 0.14 vs 1.7 ± 0.02). Adjusted logistic regression models examining the associations between ACE score and BED at two-year follow-up are presented in Table 2. One ACE (adjusted odds ratio [aOR] 3.48, 95% confidence interval [CI] 1.11–10.89), two ACEs (aOR 3.88, 95% CI 1.28–11.74), and three or more ACEs (aOR 8.94, 95% CI 3.01–26.54) were all associated with higher odds of BED at two-year follow-up. When stratified by types of ACEs, history of household mental illness (aOR 2.18, 95% 1.31–3.63), household violence (aOR 2.43, 95% CI 1.42–4.15), and criminal household member (aOR 2.14, 95% CI 1.23–3.73) were most associated with BED at two-year follow-up.

**Table 2:** Associations between adverse childhood experiences (ACEs) and binge-eating disorder (BED) at two-year follow-up in the Adolescent Brain Cognitive Development Study

	BED	
	Adjusted^a^	
	OR (95% CI)	p
Number of ACEs		
0	REF	
1	3.48 (1.11–10.89)	0.032
2	3.88 (1.28–11.76)	0.016
3 +	8.94 (3.01–26.54)	< 0.001
ACE subtypes		
Physical abuse	0.16 (0.01–2.37)	0.181
Sexual abuse	0.94 (0.11–8.15)	0.953
Emotional neglect	0.82 (0.16–4.30)	0.811
Physical neglect	1.78 (0.84–3.79)	0.135
Household substance use	1.16 (0.74–1.84)	0.516
Household divorce or separation	0.92 (0.48–1.73)	0.790
Household mental illness	**2.18 (1.31–3.63)**	**0.003**
Household violence	**2.43 (1.42–4.15)**	**0.001**
Criminal household member	**2.14 (1.23–3.73)**	**0.007**

Bold indicates p < 0.05

^a^Covariates: race/ethnicity, sex, household income, parent education, site, and baseline BED

## Discussion

In this population-based, demographically diverse sample of 10,145 9–14 year-old early adolescents in the US, the current study found that a greater number of ACEs was associated with BED in a dose-dependent manner, even after adjusting for confounders, including baseline BED. This finding confirms our hypothesis. The specific types of ACEs most strongly associated with BED at two-year follow-up were history of household mental illness, household violence, and a criminal household member.

These findings are congruent with prior research examining the relationship between ACEs and BED. However, whereas previous evidence has been confined to mostly cross-sectional clinical samples of adults receiving eating disorder treatment [11–13, 16], this study adds to the existing literature by: 1) using a large, national prospective cohort design with two-year-follow-up, 2) incorporating DSM-5 diagnosis of BED in children and adolescents, an important developmental period when long-term health behaviors are established, and 3) examining the specific types of ACEs most strongly associated with BED.

ACEs have been previously linked to several negative emotional, cognitive, and behavioral outcomes, which may help elucidate the mechanisms through which increased ACE score predisposes children and adolescents to develop BED [34]. For example, previous studies have found associations between higher levels of ACEs and negative urgency, which involves acting impulsively in times of extreme distress, alongside diminished inhibitory control [35]. Psychological models aiming to elucidate the pathways driving binge-eating behaviors describe how low self-esteem, negative affect, and difficulty with emotional regulation play a major role in their development [36], all of which have been demonstrated as psychological sequelae of ACEs [37–39]. Trauma may also have impacts on reward and inhibitory control networks [40], which has been implicated in pre-adolescent BED and in adult binge-type eating disorders [41, 42]. In a national sample of adult women, disinhibition was high in those with binge-type eating disorders, but even greater in those with comorbid PTSD, implicating the additional role of trauma on impulsivity [42]. Dissociation and depressive features associated with ACEs may further exacerbate the frequency and duration of binge-eating episodes and lead to the feelings of distress required for the diagnosis of BED [43–45]. Of note, in sensitivity analyses adjusting for baseline diagnosis of major depressive disorder, generalized anxiety disorder, and PTSD, the associations between higher number of ACEs with BED as well as specific ACE types with BED, remained unchanged.

The current study found that reported household mental illness, household violence, and having a criminal household member were all associated with BED in early adolescence. It is unclear why these specific ACEs appear to have a stronger association with BED than others. Prior studies have also attempted to examine whether certain ACEs serve as risk factors for specific eating disorders, but have reported mixed findings[11–14, 16, 46]. For instance, while some studies have found associations between different forms of childhood abuse with BED [16], others have focused on the role of neglect, such as childhood food neglect and emotional neglect [46]. The findings in the present study may reflect differences in prevalence of ACEs in different samples and different age groups. The inconsistent results underscore the need for further investigation into the role that specific ACEs may have in the development of BED.

Interestingly, household mental illness, household violence, and having a criminal household member all fall into the household dysfunction category of ACEs. This relationship may indicate a unique association between the role of the household unit and the development of binge-eating symptoms and subsequently, BED. In a community-based, case–control study of young adults with BED compared to healthy controls, parental depression was a major risk factor for BED [47]. The presence of familial and household relationships represents a central component for the emotional and developmental wellbeing of adolescents [48]. Higher familial functioning and parental support, which may not always be possible in situations involving household violence and criminal household members, have been shown to be protective of disordered eating behaviors [49]. Thus, strained relationships with family in the setting of household dysfunction may predispose individuals to BED.

Despite the strengths of the study, which include utilizing a large, diverse, and national cohort and a prospective study design, important limitations must be noted. First, although covariates were based on prior literature and theory, residual confounding remains possible. Second, given the observational study design, we cannot establish causality. Additionally, the ABCD study did not assess the severity of ACEs and also did not include questions about emotional abuse; thus, we were only able to examine nine out of the original ten ACEs that have been previously categorized [2, 4]. Furthermore, the evaluation of ACEs only determined exposure to different types of trauma, rather than the number of actual traumatic occurrences, which can be an important source of variance. This study also did not investigate recently expanded ACEs [50], which include living in foster homes, bullying, and experiencing racism. Finally, measures of BED were based on parent self-report, which may be subject to reporting bias. Although parent and child reports of binge eating have previously been shown to have low concordance [51, 52], parents are especially important reporters for EDs in this age range [53], since young children may have less insight regarding their eating behaviors [54].

## Conclusion

ACEs remain a high-priority public health issue in the US. The Healthy People 2030 initiative recently added new objectives to reduce the prevalence of ACEs in young adults. Addressing the burden of ACEs and EDs such as BED is critical given the high prevalence of ACEs and the negative outcomes associated with EDs. Although there is limited evidence that universal screening of ACEs improves the identification of childhood adversity [6], clinicians may consider ACEs in those at risk for EDs. The findings from the current study highlight the need to consider ACEs and provide trauma-focused care when evaluating and treating children and adolescents with BED, which may improve the outcomes and lived experiences of those affected [55].

## Supplementary Information

**Additional file 1: Supplemental Table 1:** Adverse Childhood Experiences (ACEs) Scale from the ABCD study.

## Data Availability

Data used in the preparation of this article were obtained from the ABCD Study (https://abcdstudy.org), held in the NIMH Data Archive (NDA).

## Abbreviations

ABCD: Adolescent brain cognitive development study
ACEs: Adverse childhood experiences
EDs: Eating disorders
BED: Binge-eating disorder
CDC: Centers for Disease Control and Prevention
PTSD: Post-traumatic stress disorder
IRB: Institutional review board
KSADS-5: Kiddie schedule for affective disorders and schizophrenia
