# Supplementary material for: Adverse childhood experiences and binge-eating disorder in early adolescents
Source: J Eat Disord. 2022 Nov 16;10:168. doi: 10.1186/s40337-022-00682-y (PMC9670461; doi:10.1186/s40337-022-00682-y)
Supplement: Supplementary file 1 — Additional file 1: Supplemental Table 1: Adverse Childhood Experiences (ACEs) Scale from the ABCD study. [file 40337_2022_682_MOESM1_ESM.docx]

**Supplemental Information**

Supplemental Table 1: Adverse Childhood Experiences (ACEs) Scale from the ABCD study

| ACE | Question | ABCD Assessment* |
| --- | --- | --- |
| Physical abuse | Shot, stabbed, or beaten brutally by a grown up in the home | KSADS-5 PTSD Module – Parent |
|  | Beaten to the point of having bruises by a grown up in the home | KSADS-5 PTSD Module – Parent |
| Sexual abuse | A grown up in the home touched your child in their privates, had your child touch their privates, or did other sexual things to your child | KSADS-5 PTSD Module – Parent |
|  | An adult outside your family touched your child in their privates, had your child touch their privates or did other sexual things to your child | KSADS-5 PTSD Module - Parent |
| Household violence | Witness the grownups in the home push, shove or hit one another | KSADS-5 PTSD Module – Parent |
|  | Family members sometimes hit each other | Environment Scale- Parent Report |
|  | Family members sometimes get so angry they throw things | Environment Scale- Parent Report |
|  | Family members sometimes hit each other | Family Environment Scale – Youth Report |
|  | Family members sometimes get so angry they throw things | Family Environment Scale – Youth Report |
| Substance abuse in the household | Has any blood relative of your child ever had any problems due to alcohol such as: marital separation or divorce, laid off or fired from work, arrests or DUIs; alcohol harmed their health; in an alcohol treatment program; suspended or expelled from school 2 or more times; isolated self from family, caused arguments or were drunk a lot?** | Family History Assessment – Parent |
| Household mental illness | Has ANY blood relative of your child ever attempted or committed suicide?** | Demographics survey – Parent |
|  | Has ANY blood relative of your child ever suffered from depression, that is, have they felt so low for a period of at least two weeks that they hardly ate or slept or couldn't work or do whatever they usually do?** | Demographics survey – Parent |
| Divorce/separation | Divorced/separated | Demographics survey – Parent |
| Criminal household member | Has ANY blood relative of your child been the kind of person who never holds a job for long, or gets into fights, or gets into trouble with the police from time to time, or had any trouble with the law as a child or an adult? | Family History Assessment – Parent |
| Emotional neglect | Believes in showing his/her love for me*** | CRPBI Acceptance Subscale – Youth |
| Physical neglect | How often do your parents/guardians know where you are?**** | Parental Monitoring Survey |
|  | If you are at home when your parents or guardians are not, how often do you know how to get in touch with them?**** | Parental Monitoring Survey |

*All ACEs data were determined through parent and adolescent responses in the baseline (2016-2018), one-year follow-up (2017-2019), and first half of two-year follow-up (2018-2019) surveys. A yes response to any of the following nine ACEs at any timepoint was counted as one-point.

**one-point given if blood relative was mother or father

***one-point if not like him/her for primary caregiver

**** one-point if never/almost never
